# Using Bakri balloon as a visceral replacement for occupying pelvic cavity in pelvic exenteration, a case report

**DOI:** 10.1016/j.ijscr.2022.107646

**Published:** 2022-09-13

**Authors:** Soheila Aminimoghaddam, Nafisseh Hivehchi, Marjan Ghaemi, Arefeh Eshghinejad, Maryam Yazdizadeh

**Affiliations:** a Firoozgar Hospital, Iran University of Medical Sciences, Tehran, Iran; b Vali-E-Asr Reproductive Health Research Center, Family Health Research Institute, Tehran University of Medical Sciences, Tehran, Iran

**Keywords:** Bakri balloon, Pelvic exenteration, Cervical cancer

## Abstract

**Introduction and importance:**

Total pelvic exenteration is the choice treatment for locally advanced or recurrent cervical cancers. However, the procedure is usually associated with serious complications. One of the most common complications is “empty pelvic syndrome”. In this case report, we described a novel method to investigate its efficacy in prevention of empty pelvic syndrome.

**Case presentation:**

A 51-year-old woman presented with recurrent cervical cancer underwent TPE after chemoradiotherapy. After removing the organs of the pelvic cavity, a silicone-made Bakri balloon was placed in there through the laparotomy incision. The balloon was removed 5 days later through the vaginal canal. She was followed for 6 months after the surgery and did not experience neither complications nor the recurrence of the cervical cancer.

**Clinical discussion:**

We intended to use a novel technique by placing a Bakri balloon in the pelvic cavity after the total pelvic exenteration. The silicone-made balloon creates an appropriate physical barrier to support colon and small intestine loops and other pelvic contents.

**Conclusion:**

Bakri balloon, which has been used to control the post-partum hemorrhage, can be a useful tool to provide a physical barrier to prevent the descending of intestinal loops and a breeding ground for reconstruction of the pelvic floor.

## Highlights

• Total pelvic exenteration is the choice treatment for locally advanced or recurrent cervical cancers.
• One of the most common complications is “empty pelvic syndrome”.
• Bakri balloon can be used as physical barrier to prevent the descending of intestinal loops.

## 1 Introduction

Cervical cancer is the most common gynecological cancer in resource-poor countries, with 270.000 deaths annually worldwide [1], [2]. Cervical cancer is mainly resulted from chronic infection by high-risk HPV [3].

Persistent infection of the cervical epithelium by high-risk HPV can lead to cervical intraepithelial neoplasia which may progress to invasive cervical cancers including squamous cell carcinoma, adenocarcinoma, adenosquamous carcinoma, and other rare histotypes [4].

Common treatments for cervical cancer include surgical resection, chemotherapy, radiotherapy, or a combination of these modalities. In addition, Molecular biology and targeted therapies are comprehensively investigated in metastatic diseases. However, it appears the therapies focusing on either the microenvironment (i.e., angiogenesis) or papillomavirus biology are unable to inhibit cervical tumor growth [5].

Total pelvic exenteration has been one of the options of choice in patients with recurrent or locally invasive cervical cancer. It is a radical surgical procedure to remove the gynecological organs and urological organs including the bladder, urethra, rectum, anus, vagina, cervix, uterus, fallopian tubes, ovaries, and sometimes the vulva [6].

TPE is a safe procedure with a roughly 0.5–2 % mortality rate [7]. Nevertheless, the morbidity rate rises up to 51 %. The complications are fistulas in the gastrointestinal tract, insufficiency of anastomosis, and wound complications in most cases [8].

It is reported that above 6 % of cases experience perineal prolapse and in 15 % of the cases fistula occurs after the surgery if a pelvic floor reconstruction method is not performed [9].

“Empty pelvis syndrome” is one of the major complications of this radical surgery, which occurs due to the vacant space in the pelvic cavity after total pelvic exenteration. The probability of sepsis, wound complications, bowel obstruction and fistulas rise because of the collection of pelvic fluid and descending of the intestinal loops in the pelvic cavity [10].

To prevent the sequalae's of the empty pelvic syndrome, several techniques have been used. However, none has gained ubiquitous acceptance [11]. Some of the methods application of artificial materials such as polypropylene mesh, degradable mesh [10], prostethic implants [12] or myocutaneous flap reconstruction [13].

Regarding the low 5-year survival rate following the abovementioned surgery (41 %) [8], in this report we put a new method into perspective by placing Bakri balloon in the pelvic cavity after total pelvic exenteration so as to see if it allows a successful management of TPE and empty pelvic syndrome. This report presents the efficacy of the Bakri balloon as a visceral replacement for occupying the pelvic cavity.

## 2 Case report

A 51-year-old Iranian female, with a history of advanced cervical cancer receiving neoadjuvant chemoradiotherapy was referred and admitted to our department. She had no positive family history and any relevant genetic information, and psychosocial history. She did not used any specific drug. After the treatment course, the involvement of her uterus was presented. After placing a nephrostomy, she underwent total pelvic exenteration by a multidisciplinary team of surgeons at Firoozgar Hospital, a tertiary care referral hospital of Iran University of Medical Sciences, because of the recurrence of the cervical cancer.

After thorough physical examination, an Abdominal pelvic CT scan was performed which revealed a mass measuring 40 ∗ 74 ∗ 132 mm which originated from the cervix and invaded to the vagina, pelvic wall, rectum, ureter, bladder and piriformis muscle.

**Fig. 1:**
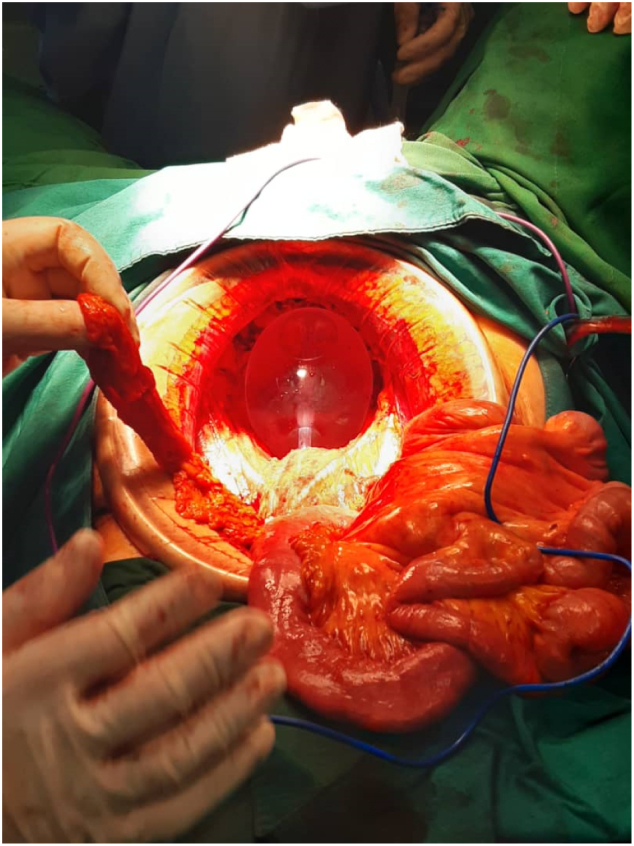
Bakri balloon was inserted immediately post-operation in pelvic exenteration.

The patient underwent a deep sedation with … and …, according to the hospital guidelines. Supine position was adopted. Following a unanimous decision made by the multidisciplinary team, total abdominal hysterectomy bilateral salpingo oophorectomy, pelvic lymphadenectomy, resection of the vagina, rectum, anus, bladder, and urethra were performed. After the procedure and rigorous homeostasis, a bakri balloon as a visceral replacement for occupying pelvic cavity was placed. The Bakri balloon was inserted through the laparotomy incision and was placed inside the pelvic cavity.The balloon was filled with sterile saline solution (maximum capacity 500 mL) to settle in the space, then the balloon was inflated gradually with sterile normal saline solution up to the minimal volume that effectively fill the cavity. Continuous traction was used by the connection of the balloon shaft to a 1-Liter intravenous fluid bag which was hanged from the end of the bed as a mechanical hemostatic agent too. Surgery last about 3 h and was performed by a fully trained gynecologic oncologist with the help of surgical oncologist and an expert urologist (Fig. 1).

The patient stayed in intensive care unit in the first day after the surgery and was discharged 5 days after the operation. The Bakri balloon was removed through the vagina 5 days after her surgery.

The patient received radiation for 3 months after her surgery, consisted of 5000 cGy. She was evaluated every month. The patient has been doing well in the absence of recurrence of the disease or any adverse effects during 6 months follow up after her surgery.

This case was reported according to Updating Consensus Surgical CAse REport (SCARE) Guidelines [14].

## 3 Discussion

Total pelvic exenteration, which is the only curative option in locally advanced and recurrent cervical cancer, is generally associated with high morbidity and mortality [15], [16]. The large eviscerated area followed by this extended surgery, predisposes to life-threatening complications such as intestinal obstruction or fistulae appearance.

In Clarke-Pearson series, the use of polyglactin film was associated with a high rate of infection, therefor it was abandoned as an alternative [17]. Lee et al., investigated the prevalence of abscess and wound complications after the usage of a degradable synthetic mesh post TPE. Half of the patients experienced collection and abscess formation [10]. Also, in a study by De-la-Noval et al. 6 patients were followed after placing a biological mesh after TPE. The patients were followed for 8.5 months, during this period several complications such as abscess formation and abdominal wall dehiscence occurred. Meanwhile, this method was not associated with perineal hernia and none of the biosynthetic mesh had to be removed. The use of biological mesh due to being high priced is also a disadvantage [9].

Wang et al. used a novel technique by maintaining the bladder peritoneum and using it as closure of the pelvic cavity and to create a mechanical support for the bowels. Despite a relatively long hospital stay, all three cases survived [11]. However, this method is not applicable in invasive cervical cancer advanced to the bladder peritoneum.

Here in our case, we intended to use a novel technique by placing a Bakri balloon in the pelvic cavity after the total pelvic exenteration. The silicone-made balloon creates an appropriate physical barrier to support colon and small intestine loops and other pelvic contents.

The limitations of our study are a relatively short-term follow-up time in solely one case. Thus, we cannot firmly conclude that this method can be considered as a successful one in preventing perineal hernias and fistulas. Having said that, our short report is so far the first one in the use of Bakri Balloon in a patient with recurrent cervical cancer. In order to confirm the efficacy of the Bakri Balloon and to identify patients who would benefit the most, larger numbers of cases and long-term follow-ups are required.

## 4 Conclusion

Bakri balloon, can be a useful tool to provide a physical barrier to prevent the descending of intestinal loops and a breeding ground for reconstruction of the pelvic floor.

## Sources of funding

This case report received no funding or sponsorships.

## Consent

The identity of the patient and her personal details are confidential.

## Ethical approval

Informed consent was received from the patient.

## CRediT authorship contribution statement

S.A.M and M.Y: Design of the work

N.H AND M.G: Drafting the manuscript

A.E Manuscript editing

## Registration of research studies

Not applicable.

## Guarantor

Dr Maryam Yazdizadeh.

## Provenance and peer review

Not commissioned, externally peer-reviewed.

## Declaration of competing interest

The authors declare no conflict of interest.
